# Modifiable Risk Factors for Alzheimer’s Disease and Related Dementias Among Middle Eastern and North African Immigrants to the United States

**DOI:** 10.1093/geroni/igae025

**Published:** 2024-02-29

**Authors:** Tiffany B Kindratt, Laura B Zahodne, Kristine J Ajrouch, Florence J Dallo

**Affiliations:** Public Health Program, Department of Kinesiology, University of Texas at Arlington, Arlington, Texas, USA; Department of Psychology, University of Michigan, Ann Arbor, Michigan, USA; Institute for Social Research, University of Michigan, Ann Arbor, Michigan, USA; Institute for Social Research, University of Michigan, Ann Arbor, Michigan, USA; Department of Sociology, Anthropology, and Criminology, Eastern Michigan University, Ypsilanti, Michigan, USA; School of Health Sciences, Oakland University, Rochester, Michigan, USA

**Keywords:** Dementia, Life-course risk factors, National Health Interview Survey, Medical Expenditure Panel Survey, MENA

## Abstract

**Background and Objectives:**

Modifiable risk factors across the life course play a role in the development of Alzheimer’s disease and related dementias (ADRD). Studies have identified racial and ethnic disparities in ADRD risk factors. Few studies have explored the epidemiology of ADRD risk among Middle Eastern and North African (MENA) Americans, largely due to their classification as White in US national health surveys. Our aim was to estimate ADRD risk factors among MENA immigrants compared to US- and foreign-born non-Hispanic White adults.

**Research Design and Methods:**

We linked cross-sectional 2000–2017 National Health Interview Survey and 2001–2018 Medical Expenditure Panel Survey data (*N* = 108 695; age ≥ 18 years). Modifiable risk factors for ADRD that were evaluated (yes or no) included less than ninth grade education, hearing loss, traumatic brain injury, hypertension, alcohol use, obesity, smoking, depressive symptoms, marital status, physical inactivity, and diabetes. Bivariate analysis and multivariable logistic regression were conducted. Regression models were adjusted by age and sex.

**Results:**

Compared to US-born White adults, MENA immigrants had higher odds of reporting less than 9th grade education (OR = 1.93; 95% CI = 1.17–3.21) and psychological health concerns (OR = 1.28; 95% CI = 1.06–1.56). Compared to foreign-born White adults, MENA immigrants had higher odds of diabetes (OR = 1.48; 95% CI = 1.06–2.08) and psychological health concerns (OR = 1.24; 95% CI = 1.01–1.54).

**Discussion and Implications:**

The findings provide the first comprehensive look at potentially modifiable risk factors for ADRD among MENA immigrants based on a life course model. Without a racial/ethnic identifier for MENA individuals on a national level, ADRD risk factors among US-born MENA adults and MENA immigrants cannot be examined. More research is needed to explore these risk factors by life stage (early, midlife, and late) to further determine ADRD risk and prevention strategies for MENA Americans.

**Translational Significance:** We investigated the burden of modifiable risk for Alzheimer’s disease and related dementias among Middle Eastern and North African (MENA) immigrants in the United States. This population is often overlooked because they are defined as part of the White racial/ethnic group by the federal government. We found a higher burden of low education, diabetes, and psychological health concerns among this group compared to US-born and foreign-born White adults. This study contributes to a line of research demonstrating that MENA health patterns differ from other White individuals. A separate identifier is needed for resources to be made available to address modifiable risk.

## Background and Objectives

In 2023, the prevalence of Alzheimer’s disease and related dementias (ADRD) was 6.7 million older adults (ages 65+ years) in the United States (US) (1). Many risk factors for ADRD are nonmodifiable: increasing age, genetics, and family history (1). Sex has also been identified as a nonmodifiable risk factor for ADRD (2). Although women tend to live longer, this risk is not fully explained by increasing age. Rather, it may be attributed to patterns of employment, marriage, and childrearing in early adulthood among other potentially modifiable risk factors (3). There is a growing body of literature demonstrating that modifiable risk factors play a major role in the development of ADRD at various points across the life course (4).

The Lancet Commission on Dementia Prevention, Intervention, and Care conducted a series of studies to synthesize the existing evidence and identify potentially modifiable risk factors that contribute to ADRD across the life course. In the latest review, 12 potentially modifiable risk factors were identified with the total population attributable fraction (PAF) of these risk factors being 40%, ranging from 1% to 8% per risk factor (4). Risk factors were identified according to the stage of life at which it is most likely to have an effect, including: less than secondary (9th grade in the US) education during early life (<45 years); hearing loss, traumatic brain injury (TBI), hypertension, excessive alcohol, and obesity during midlife (45–64 years); and smoking, depression, social isolation, physical inactivity, air pollution, and diabetes during late life (65+ years) (4). For example, if limited education during early life was removed as a risk factor, we would see a 7% reduction in dementia cases (4). This is the second largest potentially modifiable risk factor for dementia. Research on educational attainment indicates that cognitive stimulation through schooling contributes to more cognitive reserve (5) and is most important prior to adolescence (~8th grade in the US); however, higher educational attainment after high school shows additional gains in cognitive function as well as cognitively stimulating activities in middle (eg, social outings, travel, physical activity, arts) and late (eg, betting, reading, playing games) life (4). Hearing loss had the highest PAF overall and its removal during midlife would result in a reduction of 8% of dementia cases. Although the mechanisms by which hearing loss contributes to cognitive decline remain unclear, research suggests that midlife hearing loss is associated with changes to the hippocampus and entorhinal cortex in the brain (4). More research on the mechanisms that contribute to dementia among adults who experience hearing loss during midlife is needed due to an increasing prevalence of hearing loss (mean age 55 years) and lack of current knowledge of how hearing aids can reduce dementia risk. During late life, the removal of depression would result in a reduction of 4% of dementia cases (4). Because a diagnosis of depression is common among dementia patients, it is unclear whether depression contributes to a greater risk or if it’s a prodromal syndrome. The research in support of viewing depression as an independent risk factor for dementia demonstrates that changes in stress hormones, hippocampal volume, and neuronal growth factors that occur during depressive periods can contribute to cognitive decline leading to dementia (5). The identification of these risk factors is critical for developing interventions for prevention. Furthermore, it is important to consider how these risk factors are experienced differently by race/ethnicity and nativity.

Few studies have provided a comprehensive look into the prevalence of modifiable risk factors for ADRD among racial/ethnic groups in the US. Lee and colleagues (2022) presented prevalence estimates for all 12 risk factors among Hispanic, non-Hispanic Asian, non-Hispanic Black, and non-Hispanic White (hereafter, reported as Asian, Black, and White) adults in the US (6). They found that Hispanic adults were significantly more likely to report lower levels of education than Whites (27.1% vs 5.5%, respectively). Asian adults were significantly less likely to report TBI (6%) and excessive alcohol (0.7%) compared to Whites (20.1% TBI, 4.2% excessive alcohol). Although not statistically significant, Black adults were more likely than White adults to report the following: hypertension (61% vs 39.8%), obesity (54.3% vs 43.5%), smoking (11.7% vs 8.4%), physical inactivity (73.2% vs 61.3%), and diabetes (37.2% vs 25.4%) (6). Other research has identified that American Indian/Alaskan Native adults have higher prevalence estimates compared to Whites for low education (20.4% vs 7.8%), hearing loss (11.8% vs 7.4%), diabetes (17.1% vs 10.8%), current smoking (29.2% vs 16.6%), depression (25.4% vs 20.6%), physical inactivity (29.0% vs 22.1%), hypertension (47.4% vs 35.8%), and obesity (42.5% vs 34.9%) (7). Although these studies identify racial/ethnic differences, they neglect intersectionality with nativity. Furthermore, previous studies did not consider the burden of ADRD risk factors among racial/ethnic groups that are not currently part of the standard federal reporting categories in the US, specifically Middle Eastern and North African (MENA) Americans. The US MENA population is rapidly aging and growing in size (8). In 1980, the MENA immigrant population in the US was 224 000, which increased to an estimated 1.2 million in 2019 (9). In 2020, 3.5 million individuals living in the US (both US-born and immigrants) reported a MENA descent (10). Further, an increasing body of literature suggests disparities from Whites in a myriad of health outcomes (11–14), including cognitive health (15–18).

A comprehensive assessment of modifiable risk factors for ADRD has not been conducted with MENA Americans because they are defined as “White” by the federal government (19). This definition makes it challenging to investigate modifiable risk factors for this group because there are limited ways to disaggregate MENA individuals from Whites in large, nationally representative data sources. Studies using data from the National Health Interview Survey (NHIS) and Medical Expenditure Panel Survey (MEPS) have identified differences in the burden of cognitive limitations and potential underdiagnosis of ADRD among MENA immigrants compared to US- and foreign-born White older adults (15,16,18). Yet, there remains a gap in the literature on the epidemiology of modifiable risk factors for ADRD among MENA adults.

To advance the field, this study leverages nationally representative data to investigate the prevalence of modifiable risk factors for ADRD among MENA immigrants compared to US- and foreign-born Whites after adjusting for age and sex.

## Research Design and Methods

### Data Collection

The sample included linked 2000–2017 NHIS and 2001–2018 MEPS household and medical condition data. The NHIS collects a wide range of health and sociodemographic information from a complex sample of individuals using face-to-face interviews at their households. A sample of NHIS participants are selected to complete the MEPS household interview to provide additional details on healthcare visits and medical conditions. Health conditions mentioned during the household interview were recorded by the interviewer and assigned ICD-9-CM (2000–2015) and ICD-10-CM (2016–2018) codes by professional coders. Only the first 3 digits of ICD-9-CM/ICD-10-CM codes were made available to ensure confidentially. For example, ICD-9-CM codes to indicate TBI include 800–854, 905, and 907. ICD-10-CM codes to indicate TBI include S02, S06, S09, and G44. Full details of the surveys, data collection, and linkage procedures are provided on the corresponding websites (20–22). For the current study, the sample was limited to 108 695 adults ages 18 years and older (*n* = 104 731 US-born White; *n* = 3 056 foreign-born White; *n* = 908 MENA immigrants).

### Measures

#### Independent variable

The independent variable was created as a combined variable of race, ethnicity, and region of birth using NHIS data. Participants selected their race from flashcards provided during the in-person interviews (eg, White) and indicated whether their ethnicity was Hispanic or Latino/a (yes/no). The NHIS public-use files include separate variables for each (eg, RACEX and ORIGIN_I). Participants were asked whether they were born in the US, including US territories overseas (yes/no). Those who were not born in the US were asked to provide a country/region of birth. The NHIS created a variable that comprises 12 world regions (REGIONBR), specifically Europe, Russia/former USSR countries, Africa, the Middle East, the US, and others. For this study, responses were collapsed into one variable to compare US-born White, foreign-born White (inclusive of adults born in Europe and Russia/former USSR), and MENA (inclusive of adults born in the Middle East and Africa) immigrants based on previous studies (16,18). We conducted a sensitivity analysis limited to Middle Eastern immigrants to ensure our findings were not affected by foreign-born White Africans born in countries outside of North Africa (eg, South Africa) similar to previous studies (15,23–25).

#### Modifiable risk factors

Out of the 12 potentially modifiable risk factors for ADRD, 11 were evaluated. Air pollution was not available using NHIS or MEPS data. Risk factors were investigated among all adults (ages 18+ years) but described by life stage that have the greatest effect on dementia risk. NHIS data were used for alcohol use, obesity, and physical inactivity because these variables were not collected consistently by the MEPS. MEPS data were used for all other risk factors.

#### Early life

Less education was measured as a modifiable risk factor during early life. The MEPS asked, “what is the highest grade or year of regular school you/person ever completed?” Response options ranged from “never attended school/kindergarten only,” through grade school (1st through 12th grade) and “five or more years of college/graduate degree” (26). Responses were dichotomized to compare the prevalence of less than secondary education (<9th grade in the US; coded yes/no) because early education is most important in building greater cognitive reserve and less is known about whether further education is protective (5,27).

#### Midlife

Hearing loss, TBI, hypertension, alcohol use, and obesity were measured as modifiable risk factors most influential during midlife. To measure hearing loss, the MEPS asked, “is anyone in this household deaf or does anyone have serious difficulty hearing?” Responses were linked to each individual participant record and a dichotomous variable was created (yes/no). To measure TBI, MEPS annual medical condition files were linked with individual household responses. Participants with one or more of the following ICD-9-CM (800-854/905/907) or ICD-10-CM (S02/S06/S09/G44) codes were classified as reporting a TBI based on previous studies (28–30). To measure hypertension, participants indicated (yes/no) whether or not they had “ever been told by a doctor or other health professional that you had hypertension, also called high blood pressure, other than during pregnancy?” To measure alcohol use, participants responded to a series of questions on the frequency and duration of their current alcohol intake. Results were collapsed into a dichotomous variable to indicate whether they were a current drinker (yes/no) as proxy for excessive drinking. To measure obesity, a variable for body mass index (BMI) was used. Participants self-reported their weight and height. A dichotomous variable was created to compare adults who were obese (BMI = 30.0+) and not obese (BMI < 30.0).

#### Late life

Smoking, psychological health, marital status (not being married was examined as a potential indicator for social isolation), physical inactivity, and diabetes were measured as modifiable risk factors most influential during late life. To measure smoking, participants responded to a series of questions on the number, frequency, and duration of smoking cigarettes. Results were collapsed into a dichotomous variable to indicate whether they are a current smoker (yes/no). To measure psychological health concerns, participants self-reported problems with anxiety or depression as measured by the EuroQol five dimensions (EQ-5D) screener for anxiety and depression (2001–2003) and score of ≥2 on the Patient Health Questionnaire (PHQ-2; 2004–2018). The PHQ-2 includes questions on decreased interest in activities and depressed mood on a scale of 0–6. Responses to the EQ-5D questions from 2001 to 2003 (no = not anxious or depressed; yes = moderately or extremely anxious or depressed) were combined with PHQ-2 responses (no = 0–1, yes = 2–6 based on previous research (31)) from 2004 to 2016 due to changes made to the MEPS survey. An indicator of social isolation was measured with a dichotomous measure of current marital status (not married/married). Although marital status is not a comprehensive measure of social isolation, a lack of or loss of a partner has been used as indictor for social relations among older adults (18,32,33). The “not married” category included those who were never married, divorced, widowed, or separated. To measure physical inactivity, participants’ self-reported (yes/no) whether they engaged in 150 minutes or more of vigorous/moderate physical activity per week. To measure diabetes, participants indicated (yes/no) whether they had “ever been told by a doctor or other health professional that you had diabetes or sugar diabetes, other than during pregnancy?”

### Statistical Analysis

Age and sex were evaluated as demographic factors. Weighted chi-square tests compared differences in demographic factors and all risk factors across all groups and for 2-group comparisons (*p* < .05). Multivariable logistic regression models estimated associations between race, ethnicity, and nativity status and each modifiable risk factor after adjusting for age and sex. First, MENA immigrants were compared to US-born Whites. Comparisons between US- and foreign-born Whites were included to demonstrate similarities with MENA immigrants. Second, MENA immigrants were compared to foreign-born Whites. Complex sample design procedures in STATA 17.2 were used for the analysis. The MEPS household person sampling weight was used. Annual weights were combined and divided by 18 to represent the total number of years combined in the analysis (34).

The NHIS and MEPS data files used in this study were publicly available; however, the linkage file to combine NHIS and MEPS data required approval by AHRQ and restricted access at either the AHRQ Data Center or a US Census Bureau Federal Statistical Research Data Center. The data were analyzed at our local Federal Statistical Research Data Center. The study used deidentified secondary data that do meet the federal definition for human subjects. Therefore, the institutional review board at the lead author’s institution deemed it not subject for review or required approval.

## Results

### Bivariate Results

Bivariate statistics are presented in Table 1. Fewer MENA immigrants were ages 65 and older (15.1%) compared to US- (21.8%) and foreign-born (25.0%) Whites (*p* < .0001). MENA immigrants were less likely to be female (44.7%) compared to US- (51.6%) and foreign-born (53.7%) Whites (*p* < .0001). The prevalence of less education was higher among MENA immigrants (3.8%) compared to US-born Whites (2.4%; *p* = .0416). The prevalence of psychological health concerns was also higher among MENA immigrants (32.5%) compared to US- (27.5%) and foreign-born Whites (28.3%; *p* = .0404). Prevalence estimates for hearing loss (3.7%), TBI (5.2%), hypertension (22.7%), alcohol use (47.4%), obesity (24%), smoking (13.5%), and not being married were lower among MENA immigrants compared to US-born Whites (all *p*s < .01; see Table 1 for US-born White results). MENA immigrants had a lower prevalence of hearing loss, hypertension, and alcohol use compared to foreign-born Whites (all *p*s < .01; see Table 1 for foreign-born White results). There were no statistically significant differences between MENA immigrants and either US- or foreign-born Whites in the prevalence of physical inactivity or diabetes (see Table 1).

**Table 1.** Bivariate Prevalence Estimates of ADRD Risk Factors Among US- and Foreign-Born Adults Ages 18 and Older, 2000–2017 NHIS/2001–2018 MEPS, *n* = 105 673

Risk Factors	A	B	C	Significant Differences^‡‡^
	US-Born	Foreign-Born		
	White % (*SE*)	White % (*SE*)	MENA % (*SE*)	
Age				
18–44 years	42.1 (0.00)	40.7 (0.01)	49.4 (0.02)	AB, BC, AC
45–64 years	36.1 (0.00)	34.3 (0.01)	35.5 (0.02)	
65+ years	21.8 (0.00)	25.0 (0.01)	15.1 (0.02)	
Female sex	51.6 (0.00)	53.7 (0.01)	44.7 (0.01)	BC, AC
Less education^*^	2.4 (0.00)	5.7 (0.01)	3.8 (0.01)	AB, AC
Hearing loss^†^	9.0 (0.00)	6.7 (0.01)	3.7 (0.01)	AB, BC, AC
TBI^‡^	8.6 (0.00)	6.5 (0.01)	5.2 (0.01)	AB, AC
Hypertension^§^	31.8 (0.00)	30.3 (0.01)	22.7 (0.02)	AB, BC, AC
Alcohol user^∥^	64.7 (0.01)	70.2 (0.02)	47.4 (0.04)	AB, BC, AC
Obesity^¶^	28.9 (0.00)	21.6 (0.01)	24.0 (0.02)	AB, AC
Current smoker^∥^	19.7 (0.00)	15.6 (0.01)	13.5 (0.02)	AB, AC
Psychological health^#^	27.5 (0.00)	28.3 (0.01)	32.5 (0.02)	AC
Not married^**^	42.4 (0.00)	34.9 (0.01)	30.4 (0.02)	AB, AC
Physical inactivity^††^	79.6 (0.00)	80.4 (0.01)	80.3 (0.02)	—
Diabetes^§^	8.6 (0.00)	7.5 (0.01)	8.6 (0.01)	—

*Notes*: ADRD = Alzheimer’s disease and related dementias; MENA = Middle Eastern or North African; MEPS = Medical Expenditure Panel Survey; NHIS = National Health Interview Survey; TBI = Traumatic Brain Injury.

^*^Less education (<9th grade, no secondary education) was determined by highest level of education reported (yes or no).

^†^Hearing loss (yes or no) was determined by self-report of any hearing difficulty, including some or serious difficulty.

^‡^TBI (yes or no) was determined if ICD-9-CM (800-854/905/907) or ICD-10-CM (S02/S06/S09/G44) codes were in medical condition files.

^§^Hypertension (yes or no) and diabetes (yes or no) were determined responses to questions asking whether a doctor or other health professional ever told the participant they had each condition.

^∥^Alcohol use (yes or no) and smoking (yes or no) were measured by report of current drinking or smoking.

^¶^Obesity was determined by self-reported body mass index of ≥30 kg/m^2^ (yes or no).

^#^Psychological health was determined by self-report of problems with anxiety or depression as measured by EQ-5D (2001–2003) or score of 2 or greater on Patient Health Questionnaire (PHQ2; 2004–2018) measuring little interest or pleasure or feeling down/depressed.

^**^Current marital status was used as an indicator for social isolation (yes or no). “Not married” responses included divorced, widowed, and separated compared to currently “Married.”

^††^Physical inactivity (yes or no) determined by self-report or current moderate to vigorous physical activity at least one half-hour 5 times a week.

^‡‡^ *p* < .05, weighted chi-square.

### Regression Analysis

Multivariable logistic regression models adjusted for age and sex are presented in Table 2. Compared to US-born Whites, MENA immigrants had statistically significant higher odds of reporting less than 9th grade education (OR = 1.93; 95% CI = 1.17–3.21) and psychological health concerns (OR = 1.28; 95% CI = 1.06–1.56). MENA immigrants had statistically significant lower odds of hearing loss (OR = 0.42; 95% CI = 0.28, 0.64), hypertension (OR = 0.67; 95% CI = 0.56–0.81), alcohol use (OR = 0.42; 95% CI = 0.31–0.56), obesity (OR = 0.79; 95% CI = 0.63–0.98), current smoking (OR = 0.60; 95% CI = 0.46–0.77), and a potential indication of social isolation from not being married (OR = 0.57; 95% CI = 0.46–0.71) compared to US-born Whites. There were no statistically significant differences in the odds of physical inactivity (OR = 1.05; 95% CI = 0.84–1.31) or diabetes (OR = 1.15; 95% CI = 0.86–1.54) among MENA immigrants and US-born Whites. Compared to foreign-born Whites, MENA immigrants had statistically significant higher odds of diabetes (OR = 1.48; 95% CI = 1.06–2.08) and psychological health concerns (OR = 1.24; 95% CI = 1.01–1.54). MENA immigrants had statistically significant lower odds of alcohol use (OR = 0.31; 95% CI = 0.22–0.44) and a potential indication of social isolation from not being married (OR = 0.76; 95% CI = 0.61, 0.96) compared to foreign-born Whites. There were no statistically significant differences in the odds of reporting less than high school education (OR = 0.84; 95% CI = 0.50–1.41), hearing loss (OR = 0.66; 95% CI = 0.43–1.02), TBI (OR = 0.79; 95% CI = 0.56–1.12), hypertension (OR = 0.82; 95% CI = 0.66–1.03), obesity (OR = 1.18; OR = 0.92–1.50), smoking (OR = 0.77; 95% CI = 0.57–1.03), or physical inactivity (OR = 1.00; 95% CI = 0.78, 1.29) among MENA immigrants and foreign-born Whites.

**Table 2.** Multivariable Logistic Regression Models Examining Odds of Potentially Modifiable Risk Factors for ADRD, Ages 18 and Older, 2000–2017 NHIS/2001–2018 MEPS, *n* = 105 673

Potentially Modifiable Risk Factors	Model 1^†^ OR (95% CI)	Model 2^‡^ OR (95% CI)
Less education^§^		
US-born non-Hispanic White	1.00	
Foreign-born non-Hispanic White	2.31 (1.90, 2.81)^*^	1.00
Foreign-born MENA	1.93 (1.17, 3.21)^*^	0.84 (0.50, 1.41)
Hearing loss^∥^		
US-born non-Hispanic White	1.00	
Foreign-born non-Hispanic White	0.64 (0.54, 0.76)^*^	1.00
Foreign-born MENA	0.42 (0.28, 0.64)^*^	0.66 (0.43, 1.02)
TBI^¶^		
US-born White	1.00	
Foreign-born White	0.74 (0.63, 0.88)^*^	1.00
Foreign-born MENA	0.59 (0.43, 0.81)^*^	0.79 (0.56, 1.12)
Hypertension^#^		
US-born non-Hispanic White	1.00	
Foreign-born non-Hispanic White	0.82 (0.73, 0.91)^*^	1.00
Foreign-born MENA	0.67 (0.56, 0.81)^*^	0.82 (0.66, 1.03)
Alcohol user^**^		
US-born non-Hispanic White	1.00	
Foreign-born non-Hispanic White	1.33 (1.13, 1.55)^*^	1.00
Foreign-born MENA	0.42 (0.31, 0.56)^*^	0.31 (0.22, 0.44)^*^
Obesity^††^		
US-born non-Hispanic White	1.00	
Foreign-born non-Hispanic White	0.67 (0.60, 0.75)^*^	1.00
Foreign-born MENA	0.79 (0.63, 0.98)^*^	1.18 (0.92, 1.50)
Current smoker^**^		
US-born non-Hispanic White	1.00	
Foreign-born non-Hispanic White	0.78 (0.67, 0.90)^*^	1.00
Foreign-born MENA	0.60 (0.46, 0.77)^*^	0.77 (0.57, 1.03)
Psychological health^‡‡^		
US-born non-Hispanic White	1.00	
Foreign-born non-Hispanic White	1.03 (0.93, 1.15)	1.00
Foreign-born MENA	1.28 (1.06, 1.56)^*^	1.24 (1.01, 1.54)^*^
Not married^§§^		
US-born non-Hispanic White	1.00	
Foreign-born non-Hispanic White	0.75 (0.67, 0.83)^*^	1.00
Foreign-born MENA	0.57 (0.46, 0.71)^*^	0.76 (0.61, 0.96)^*^
Physical inactivity^∥∥^		
US-born non-Hispanic White	1.00	
Foreign-born non-Hispanic White	1.05 (0.93, 1.17)	1.00
Foreign-born MENA	1.05 (0.84, 1.31)	1.00 (0.78, 1.29)
Diabetes^#^		
US-born non-Hispanic White	1.00	
Foreign-born non-Hispanic White	0.78 (0.65, 0.93)^*^	1.00
Foreign-born MENA	1.15 (0.86, 1.54)	1.48 (1.06, 2.08)^*^

*Notes*: ADRD = Alzheimer’s disease and related dementias; MENA = Middle Eastern or North African; MEPS = Medical Expenditure Panel Survey; NHIS = National Health Interview Survey; TBI = Traumatic Brain Injury.

^*^ *p* < .05 denotes statistical significance.

^†^Model 1 adjusted for age and sex. Reference group is US-born Whites.

^‡^Model 2 adjusted for age and sex. Reference group is foreign-born Whites.

^§^Less education (<9th grade, no secondary education) was determined by highest level of education reported (yes or no).

^∥^Hearing loss (yes or no) was determined by self-report of any hearing difficulty, including some or serious difficulty.

^¶^TBI (yes or no) was determined if ICD-9-CM (800-854/905/907) or ICD-10-CM (S02/S06/S09/G44) codes were in medical condition files.

^#^Hypertension (yes or no) and diabetes (yes or no) were determined responses to questions asking whether a doctor or other health professional ever told the participant they had each condition.

^**^Alcohol use (yes or no) and smoking (yes or no) were measured by report of current drinking or smoking.

^††^Obesity was determined by self-reported body mass index of ≥30 kg/m^2^ (yes or no).

^‡‡^Psychological health was determined by self-report of problems with anxiety or depression as measured by EQ-5D (2001–2003) or score of 2 or greater on Patient Health Questionnaire (2004–2018) measuring little interest or pleasure or feeling down/depressed.

^§§^Current marital status was used as a potential indicator for social isolation (yes or no). “Not married” responses included divorced, widowed, and separated compared to currently “Married.”

^∥∥^Physical inactivity (yes or no) determined by self-report or current moderate to vigorous physical activity at least one half-hour 5 times a week.

Results from the sensitivity analysis with Middle Eastern immigrants are presented in Supplementary Table 1. All confidence intervals overlapped when we compared results among Middle Eastern and MENA immigrants, which demonstrates our findings are not biased from Whites born in African countries outside of the MENA region.

## Discussion and Implications

In this study, we provided a comprehensive overview of potentially modifiable risk factors for ADRD among MENA immigrants compared to US- and foreign-born Whites. We found both risk and protective risk factors for MENA immigrants compared with both groups. This is the first study to provide a comprehensive investigation into modifiable ADRD risk among MENA Americans. An overview of key findings in comparison to previous studies is provided in the following sections.

First we found that MENA immigrants were more likely to report less than secondary education compared to US-born Whites. If this risk factor is eliminated, it represents a 7% reduction in ADRD prevalence on a population level, which is one of the greatest modifiable risk factors identified by previous research (4). Several studies assessing the health of MENA Americans have included an assessment of education as a contributing factor to the association between race/ethnicity and health conditions using nationally representative data.

Most studies have made comparisons by highest level of education, with less than high school (12th grade education) as the lowest level and Bachelor’s degree or more education as the highest level. Other studies have reported that 13.6%–23.4% of MENA immigrants ages 18 and older have less than high school education compared to 10.8%–27.23% of US-born Whites (14,24,35–39). Because the sample in this study includes adults who are already 18 and older, it would not be possible to modify their level or type of early-life education. Rather, the results can be used to inform the design of interventions to account for adults’ existing lower levels of education, target other risk factors more prevalent in this population, such as psychological health concerns, and/or demonstrate the potential utility of early-life educational interventions for future generations.

Second we found that MENA immigrants were more likely to report psychological health concerns compared to both US- and foreign-born Whites. If this risk factor is eliminated during later life, it represents a 4% reduction in ADRD prevalence on a population level (4). Our finding contributes to a growing body of literature that identifies this disparity using national, clinic, and community-based samples (23,35,40). Dallo and colleagues (13) reported that MENA immigrants had greater odds of reporting serious psychological distress (SPD) compared to both US- (OR = 1.76; 95% CI = 1.01–3.04) and foreign-born Whites (OR = 2.43; 95% CI = 1.15–5.14). SPD was measured using the Kessler K6 Scale, which is a 6-item validated scale (0 = none of the time to 5 = all of the time) included in the NHIS that measures how often adults experience (1) sadness, (2) nervousness, (3) restlessness, (4) helplessness; (5) effortless, and (6) worthlessness during the past 30 days. Scores for each question were combined and total scores greater than or equal to 13 indicate SPD (41). Other research has shown that factors associated with SPD among MENA immigrants included sex, seeing a mental healthcare provider, and obesity. MENA women, those who saw a mental healthcare provider, and those who were obese had higher odds of SPD compared to men, those who did not seek mental healthcare, and those with a healthy weight (35). Programs designed to increase social engagement and awareness of key community resources could help mitigate risk in this population. In a longitudinal study of depression among immigrant MENA women, changes in the levels of social support from friends were a factor contributing to depression after 18 months (42). Another study by Goodkind and colleagues (2020) reported on an intervention designed to reduce postmigration stressors by providing social support and making information on key community resources more readily available for recent refugees from the MENA region (Afghan, Iraqi, and Syrian). Results demonstrated a reduction in depression and anxiety after 12 months. The results highlight the importance of making funding available for developing and maintaining these programs (43). Programs to improve screening and follow-up for depression can also help lower the burden of psychological health concerns. However, research conducted in Michigan, which has the second largest population of MENA Americans, has demonstrated that MENA Americans are less likely to be screened and receive follow-up care from a behavioral specialist than Whites (44). With the stigma associated with seeking and participating in mental health counseling (45), and without a separate ethnic identifier to capture psychological health among both US- and foreign-born MENA Americans on a national level, baseline estimates for screening, diagnosis, and follow-up care will remain unknown.

Third we found that MENA immigrants were more likely to report having diabetes compared to foreign-born Whites, but there was no difference when compared to US-born Whites. If this risk factor is eliminated during later life, it represents a 1% reduction in ADRD prevalence on a population level (4). Foreign-born Whites had lower odds of diabetes compared to US-born Whites, which is consistent with other research (37). Although our purpose was not to compare foreign-born to US-born Whites, it is important to acknowledge this result for validity and reliability purposes. Our findings are similar to studies that have compared diabetes among MENA immigrants and US-born Whites using nationally representative data. Using data from the NHIS, Dallo and Borrell (2006) did not find a difference in the prevalence in diabetes among MENA immigrants compared to US-born Whites (36). A follow-up study found similar results when comparing MENA immigrants to US-born Whites, but identified that MENA immigrants have a higher prevalence of diabetes in combination with other diseases compared to both US- and foreign-born Whites. Dallo and Kindratt (2016) found that the prevalence of comorbid diabetes (>1 other chronic condition) was 8% among MENA immigrants compared to 6% among US-born and 5% among foreign-born Whites (37). Future studies examining MENA health should be mindful that MENA Americans with diabetes may have an increased burden of other chronic conditions given that other studies have reported high levels of comorbidity ranging from 59% to 97% regardless of race/ethnicity and nativity (46,47).

Overall, the findings from this study are consistent with previous research using nationally representative data to capture ADRD risk factors among MENA immigrants but may differ from the findings obtained from state or local samples. Without support for an ethnic identifier on a national level, the ability to determine baseline estimates for each of these risk factors is limited. In addition to the NHIS, the American Community Survey is the only other survey that allows for nationally representative data to be collected on MENA Americans by using questions on ancestry. However, the only modifiable risk factor for ADRD that can be measured is education. In January 2023, the Office of Management and Budget released a proposal for changing the way that race and ethnicity data are collected (48). The proposal included a recommendation for adding a MENA checkbox so individuals whose heritage is from this region can be identified and resources be made available. The proposal was posted online for 90 days and over 21 000 comments were received. In an initial review of 6 700 comments received, over 70% mentioned the addition of a MENA checkbox and of those, 99% supported its inclusion and 29% mentioned support for health reasons (49).

### Strengths and Limitations

The ability to disaggregate MENA immigrants from Whites is a strength of this study given the lack of an ethnic identifier for MENA Americans in nationally representative data sets. Linking multiple years of 2 nationally representative data sources is an additional strength. Methods for disaggregating and linking NHIS and MEPS data to uncover MENA health estimates have been used previously to study older adults (65+ years) (16,18). This is the first study to use this method to capture MENA health for adults ages 18 and older. Linking NHIS and MEPS data allows us to assess a broader range of health outcomes that could be measured by using NHIS data alone. This study used MEPS medical condition files to calculate the first prevalence estimates for TBI among MENA immigrants. It is important to note that our findings are cross-sectional. Risk factors were measured in a sample that represented all years of adulthood but were not queried by the stage of life when the risk for dementia may be most potent and/or modifiable. A potential limitation is that our regression models were only adjusted for age and sex. To provide a comprehensive outlook of all potentially modifiable risk factors, we aimed to adjust for the same variables for each outcome. With not being married being tested as an indicator of social isolation and low education being examined as outcomes, those variables were unable to be adjusted for in other regression models. Furthermore, the pathways for other factors such as health insurance coverage were not appropriate to adjust for when examining outcomes such as low education or physical inactivity. Estimates for US-born MENA adults could not be calculated because the NHIS does not have a race or ethnicity identifier for MENA Americans. Some US-born MENA adults may be included in the US-born White category and health burdens may be different if this group was not masked under the US-born White group. Another limitation is that NHIS and MEPS only collect data in English and Spanish. This is an enormous barrier to collecting data on MENA health. A recent US Census report found that Arabic was the fastest-growing language spoken in the US. When comparing data from 2006–2010 and 2017–2021, there was a roughly 70% increase in households with Arabic spoken at home (50). In the aforementioned review of initial comments posted regarding the potential addition of a MENA checkbox on the US Census and other federal forms, 45% of those in support of a MENA checkbox mentioned the need for resources to be made available for linguistic services (49). Without linguistic services other than Spanish in the NHIS and MEPS, we may be missing participants and therefore underestimating modifiable risks among monolingual Arabic speakers.

## Conclusions

The findings from this study provide the first comprehensive investigation into modifiable risk for ADRD among MENA immigrants and reveal evidence for both risk and resilience in this group. Without a racial/ethnic identifier for MENA individuals nationally, ADRD risk factors among US-born MENA adults cannot be examined. More research is needed to explore these risk factors by life stage (early/midlife/late) and potency to further determine ADRD risk and prevention strategies for MENA Americans.

## Supplementary Material

igae025_suppl_Supplementary_Table

## Data Availability

The publicly available data from the National Health Interview Survey and Medical Expenditure Panel Survey are available from the National Center for Health Statistics and Agency for Healthcare Research and Quality websites. Researchers interested in replicating the findings from this study must request to use the restricted data files from the Agency for Healthcare Research and Quality.
