# Supplementary material for: Modifiable Risk Factors for Alzheimer’s Disease and Related Dementias Among Middle Eastern and North African Immigrants to the United States
Source: Innov Aging. 2024 Feb 29;8(3):igae025. doi: 10.1093/geroni/igae025 (PMC10960626; doi:10.1093/geroni/igae025)
Supplement: igae025_suppl_Supplementary_Table [file igae025_suppl_supplementary_table.docx]

*Innovation in Aging* Supplementary Material: Tiffany B. Kindratt, Laura B. Zahodne, Kristine J. Ajrouch, & Florence J. Dallo. Modifiable risk factors for Alzheimer’s disease and related dementias among Middle Eastern and North African immigrants to the United States.

**Supplemental Table 1.** Multivariable logistic regression models examining odds of potentially modifiable risk factors for ADRD among foreign-born Middle Eastern adults compared to US- and foreign-born Whites, ages 18 and older, 2000-2017 NHIS/2001-2018 MEPS, n = 108,466.

| **Potentially Modifiable Risk Factors** | **Model 1^a^**  **OR (95% CI)** | **Model 2^b^**  **OR (95% CI)** |
| --- | --- | --- |
| **Less Education^c^** |  |  |
| US-born non-Hispanic White | 1.00 |  |
| Foreign-born non-Hispanic White | 2.31 (1.90, 2.81)* | 1.00 |
| Foreign-born Middle Eastern | 2.57 (1.51, 4.40)* | 1.11 (0.64, 1.92) |
| **Hearing Loss^d^** |  |  |
| US-born non-Hispanic White | 1.00 |  |
| Foreign-born non-Hispanic White | 0.64 (0.54, 0.76)* | 1.00 |
| Foreign-born Middle Eastern | 0.45 (0.28, 0.73)* | 0.71 (0.43, 1.16) |
| **TBI^e^** |  |  |
| US-born White | 1.00 |  |
| Foreign-born White | 0.74 (0.63, 0.88)* | 1.00 |
| Foreign-born Middle Eastern | 0.58 (0.41, 0.84)* | 0.79 (0.53, 1.16) |
| **Hypertension^f^** |  |  |
| US-born non-Hispanic White | 1.00 |  |
| Foreign-born non-Hispanic White | 0.82 (0.73, 0.91)* | 1.00 |
| Foreign-born Middle Eastern | 0.57 (0.45, 0.71)* | 0.70 (0.54, 0.89)* |
| **Alcohol User^g^** |  |  |
| US-born non-Hispanic White | 1.00 |  |
| Foreign-born non-Hispanic White | 1.33 (1.13, 1.55) | 1.00 |
| Foreign-born Middle Eastern | 0.38 (0.27, 0.54)* | 0.29 (0.20, 0.42)* |
| **Obesity^h^** |  |  |
| US-born non-Hispanic White | 1.00 |  |
| Foreign-born non-Hispanic White | 0.67 (0.60, 0.75)* | 1.00 |
| Foreign-born Middle Eastern | 0.73 (0.56, 0.95)* | 1.09 (0.82, 1.46) |
| **Current Smoker^g^** |  |  |
| US-born non-Hispanic White | 1.00 |  |
| Foreign-born non-Hispanic White | 0.78 (0.67, 0.90)* | 1.00 |
| Foreign-born Middle Eastern | 0.69 (0.52, 0.92)* | 0.89 (0.64, 1.22) |
| **Psychological Health^i^** |  |  |
| US-born non-Hispanic White | 1.00 |  |
| Foreign-born non-Hispanic White | 1.03 (0.93, 1.15) | 1.00 |
| Foreign-born Middle Eastern | 1.26 (1.01, 1.58)* | 1.22 (0.95, 1.56) |
| **Not Married^j^** |  |  |
| US-born non-Hispanic White | 1.00 |  |
| Foreign-born non-Hispanic White | 0.75 (0.67, 0.83)* | 1.00 |
| Foreign-born Middle Eastern | 0.57 (0.45, 0.73)* | 0.76 (0.59, 0.98)* |
| **Physical Inactivity^k^** |  |  |
| US-born non-Hispanic White | 1.00 |  |
| Foreign-born non-Hispanic White | 1.05 (0.93, 1.17) | 1.00 |
| Foreign-born Middle Eastern | 1.05 (0.80, 1.37) | 1.00 (0.75, 1.34) |
| **Diabetes^f^** |  |  |
| US-born non-Hispanic White | 1.00 |  |
| Foreign-born non-Hispanic White | 0.78 (0.65, 0.93)* | 1.00 |
| Foreign-born Middle Eastern | 1.05 (0.73, 1.50) | 1.35 (0.90, 2.01) |

*Abbreviations. ADRD = Alzheimer’s disease and related dementias; MENA = Middle Eastern or North African; MEPS =* Medical Expenditure Panel Survey; TBI = Traumatic Brain Injury

*p<.05 denotes statistical significance.

^a^Model 1 adjusted for age and sex. Reference group is US-born Whites.

^b^Model 2 adjusted for age and sex. Reference group is foreign-born Whites.

^c^Less education (<9^th^ grade, no secondary education) was determined by highest level of education reported (yes or no).

^d^Hearing loss (yes or no) was determined by self-report of any hearing difficulty, including some or serious difficulty.

^e^TBI (yes or no) was determined if ICD-9-CM (800-854/905/907) or ICD-10-CM (S02/S06/S09/G44) codes were in medical condition files.

^f^Hypertension (yes or no) and diabetes (yes or no) were determined responses to questions asking whether a doctor or other health professional ever told the participant they had each condition.

^g^Alcohol use (yes or no) and smoking (yes or no) were measured by report of current drinking or smoking.

^h^Obesity was determined by self-reported body mass index (BMI) of ≥30 kg/m^2^ (yes or no).

^i^Psychological health was determined by self-report of problems with anxiety or depression as measured by EQ-5D (2001-2003) or score of 2 or greater on Patient Health Questionnaire (PHQ2) (2004-2018) measuring little interest or pleasure or feeling down/depressed.

^j^Current marital status was used as a potential indicator for social isolation (yes or no). “Not Married” responses included divorced, widowed, and separated compared to currently “Married.”

^k^Physical inactivity (yes or no) determined by self-report or current moderate to vigorous physical activity at least one half-hour five times a week.
